# Supplementary material for: Enhancement of zebrafish sperm production via a large body-sized surrogate with germ cell transplantation
Source: Commun Biol. 2023 Apr 14;6:412. doi: 10.1038/s42003-023-04800-7 (PMC10104805; doi:10.1038/s42003-023-04800-7)
Supplement: Supplementary file 2 — Supplementary Information [file 42003_2023_4800_MOESM2_ESM.pdf]

Enhancement of zebrafish sperm production via a large body-sized surrogate with germ cell transplantation

Rigolin Nayak* [a], Roman Franěk [a, b], Radek Šindelka [c], Martin Pšenička [a]

[a] The University of South Bohemia in Ceske Budejovice, Faculty of Fisheries and Protection of Waters, South Bohemian Research Center of Aquaculture and Biodiversity of Hydrocenoses, Zatisi 728/II, 389 25 Vodnany, Czech Republic.

[b] Department of Genetics, The Silberman Institute, The Hebrew University of Jerusalem, Jerusalem, Israel.

[c] Laboratory of Gene Expression, Institute of Biotechnology, BIOCEV, Prumyslova 595, 252 50 Vestec, Czech Republic

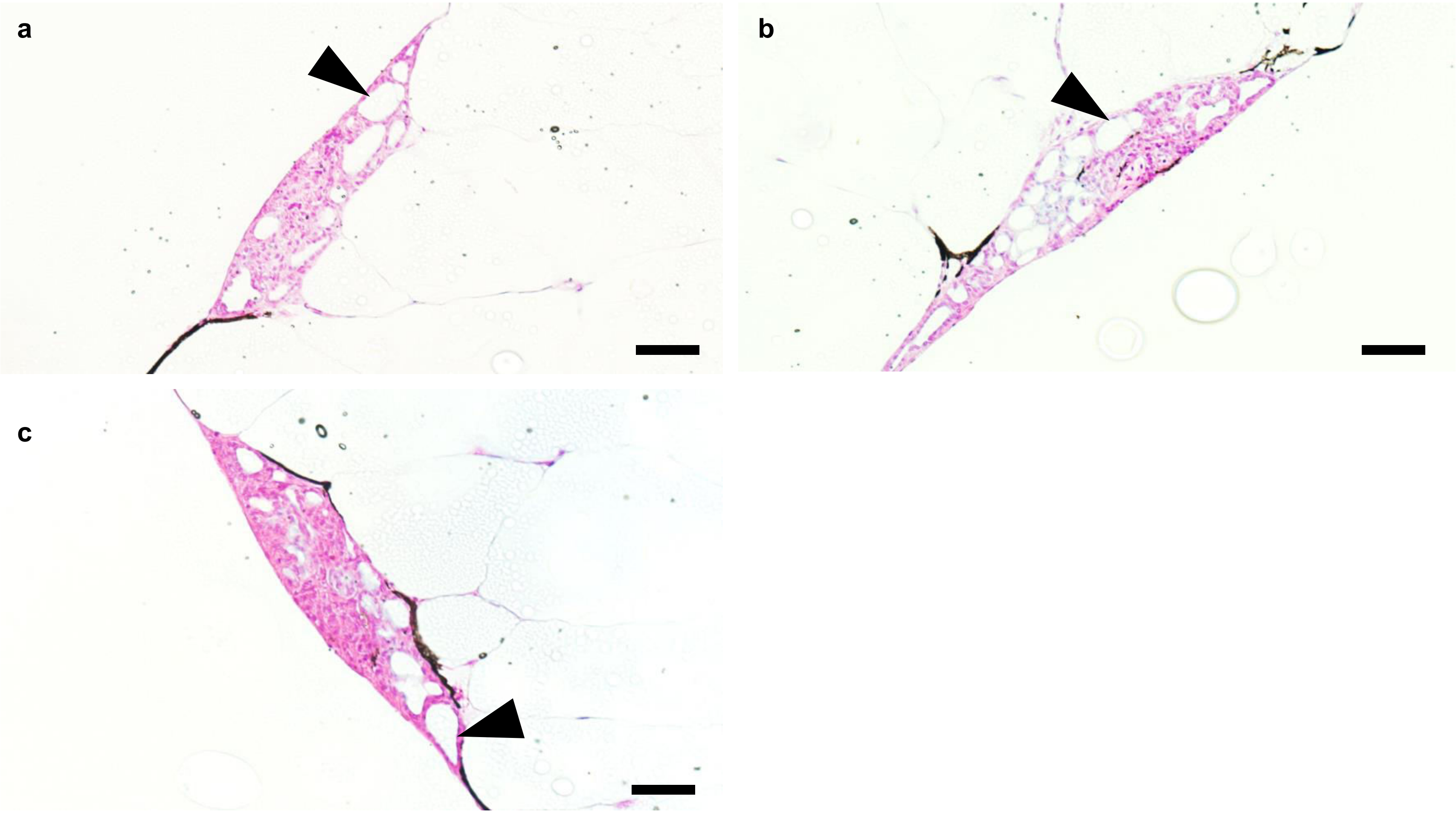

Supplementary Figure 1. Histological sections sterile giant danio gonad. Underdeveloped gonads in other sterile giant danio shows the testis-like structure, the empty lumen are indicated by black arrowheads **a**) Male-1, **b**) Male-2 and **c**) Male-3. Scale bars – 100µm

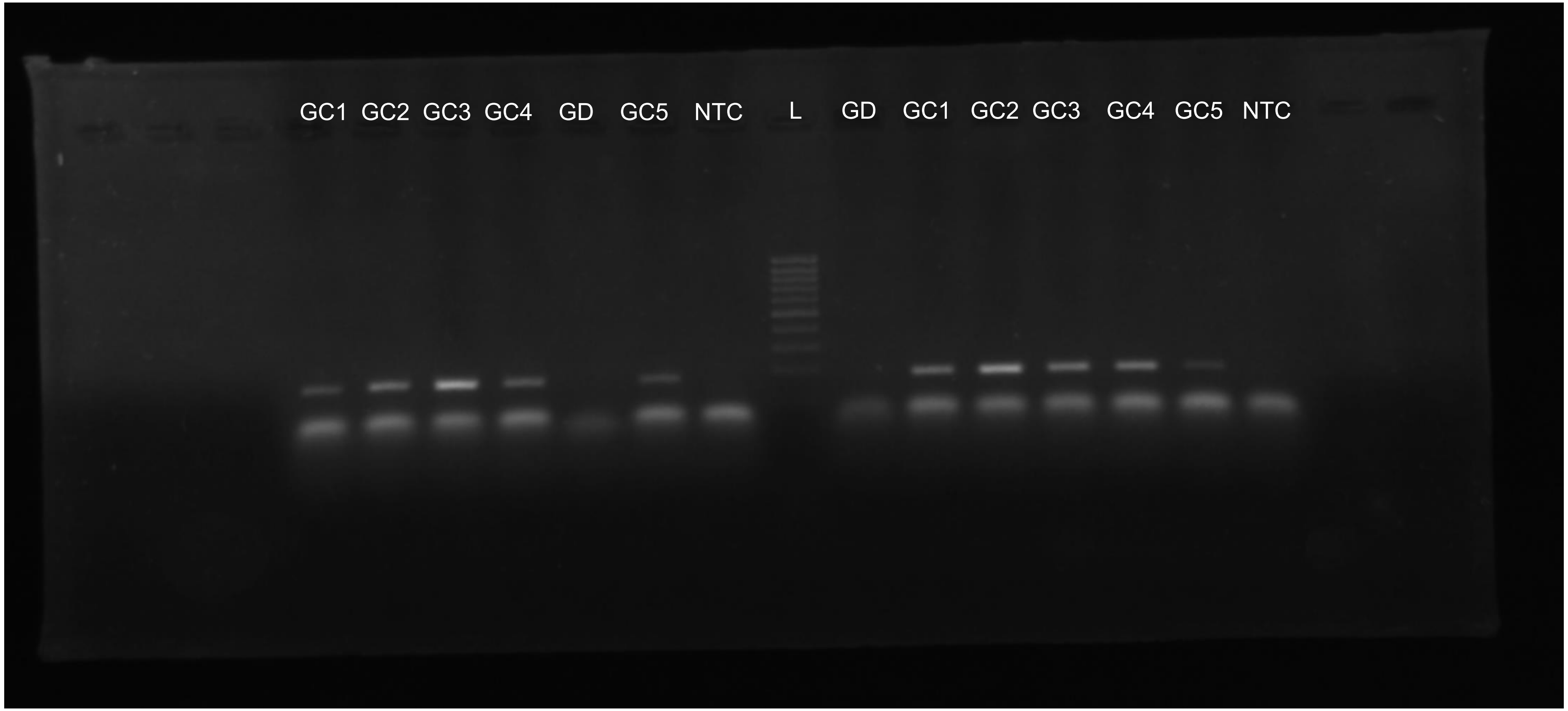

Supplementary Figure 2. Uncropped and unedited gel image for GFP gene specific PCR. Donor-derived sperm from five germline chimera were subjected to GFP- specific PCR amplification. This gene specific amplification was repeated two times for each sample. Germline chimera (GC1-GC5) shows positive amplification for the GFP gene, and the product length is 187bp loaded on 1.5% agarose gel. GD- sperm from giant danio males shows no PCR amplification, L-100bp DNA marker, and NTC is no template control.

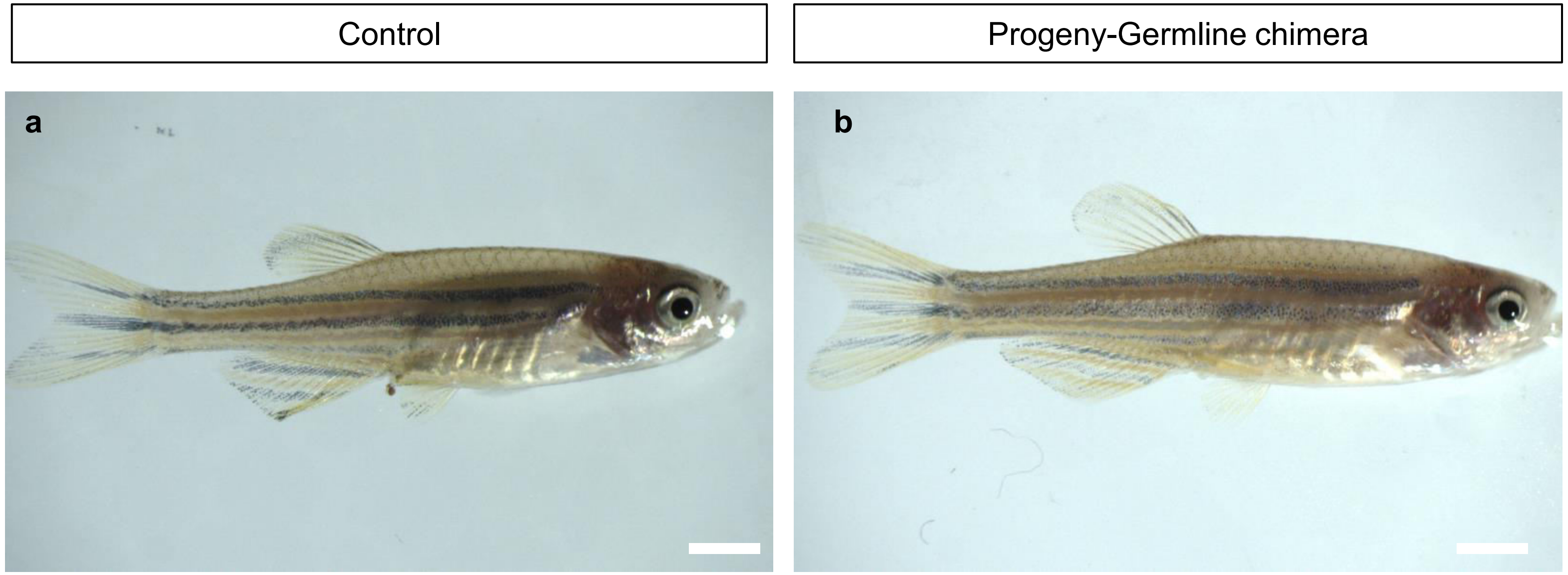

Supplementary Figure 3. Morphology of the donor-derived offspring. **a)** Two months old progeny from AB control group. **b)** progeny produced by germline chimera sperm and AB female oocytes. Scale bars – 1mm

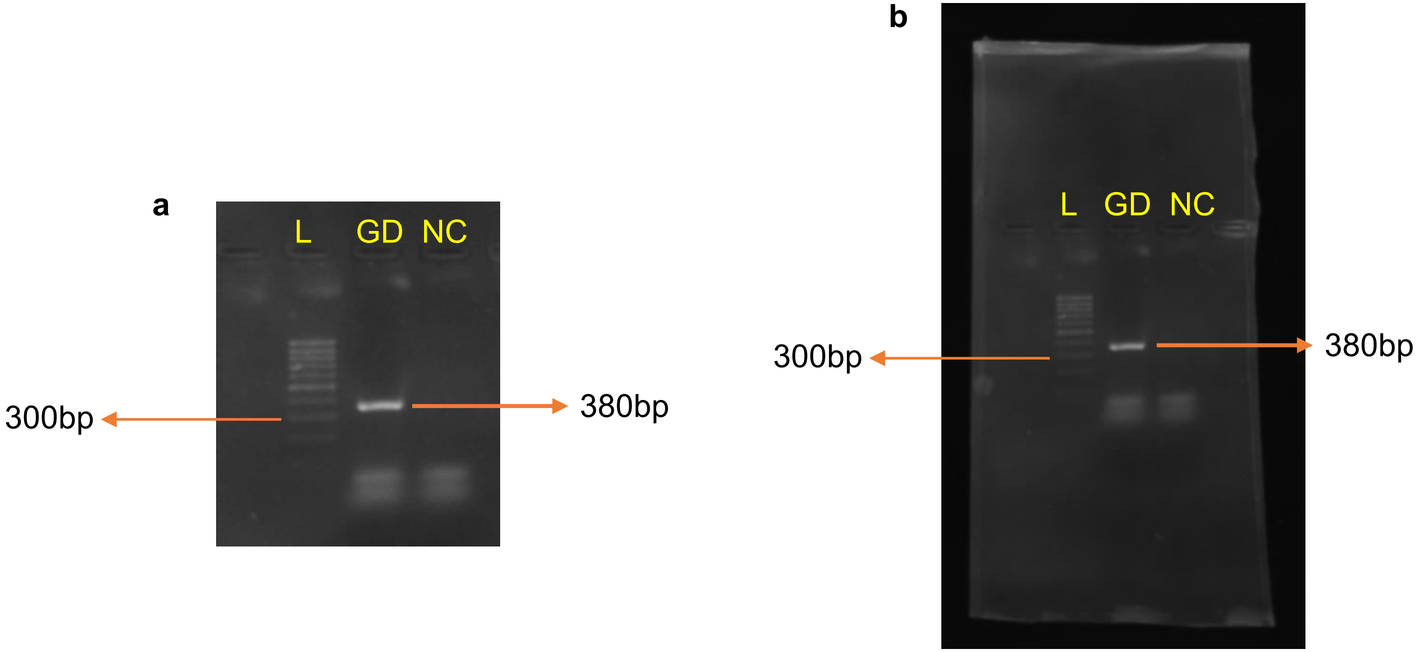

Supplementary Figure 4. Gel image for giant danio dead-end gene amplification. a) The 380bp amplicon is the dead-end gene amplified from the gonadal tissue of adult giant danio. **b**) Uncropped and unedited gel image for Figure **a** (left). GD-Giant danio, NC- No template control and L- 100bp DNA marker on 2% agarose gel.

```
Oryzias         ------------------------------------------------------------ 0
Oncorhynchus    ------------------------------------------------------------ 0
Carassius       ------------------------------------------------------------ 0
Cyprinus        ------------------------------------------------------------ 0
Devario         -----------------------------------------------------------G 1
Danio           CTTTAATGACCTTTTCTTGACTTTTCCACCAATTTACAGGTGTGTCTATCATCATCATCA 60

Oryzias         ---------------------------ATGGACAATCAGAGCAAGGTGGTGAACTTGGAG 33
Oncorhynchus    ------------------------ATGGAGGAGCGTTCAAGTCAGGTGTTGAACCCGGAG 36
Carassius       ---------------------ATGGAGGGACAGCAGCTACAGCAGGTTTTGAACCCGCAG 39
Cyprinus        ---------------------ATGGAGGGACAGCAGCTACAGCAGGTTTTGAACCCACAC 39
Devario         GAGATGCTCGCAGACATGGA---GGCTCAACGGCATTTACAGCAGATTCTGAACCCGGTC 58
Danio           CAGATGGTCGGAGACATGGATGCCCAGCAGCAGGAGCTTCAGCAGATTCTGAACCCGCAG 120
                                                           ** *  *****

Oryzias         CGGGTCCAGGCACTTCAGGCCTGGGTCAAGTCCACCAATACCAAACTGACCCAGGTTAAC 93
Oncorhynchus    CGACTGAAGGCGCTGGAGATGTGGCTGCAGGAGACTGACGTCAAACTGACCCAAGTCAAT 96
Carassius       AGACTGAAATCACTACAAGAATGGATGCAGAAAAGCTCAGTCACTTTAACACAGGTCAAT 99
Cyprinus        AGACTGAAGTCACTACAAGAATGGATGCAGAAAAGCTCAATCACTTTAACACAGGTCAAT 99
Devario         AAAGTGAAGTCACTGCAGGAATGGATGCAGAAAAACTCCATCAATTTAACCCAGGTCAAC 118
Danio           AAACTCAAGTCTCTGCAGGAATGGATGCAGAGGAACTCCATCACTTTAACCCAAGTCAAT 180
                    *  *  * **  *    *** *  **   *       **   * ** ** ** **

Oryzias         GGCCAGAGGAAGTATGGAGGGCCACCTGATGTGTGGGACGGTCCCCCACCTGGGGCGCGC 153
Oncorhynchus    GGCCAGAGAAAATATGGAGGTCCACCTGATGATTGGCTTGGCGCCCCCCCTGGGCCGGGC 156
Carassius       GGGCAGAGGAAATATGGTGGTCCTCCTGTTGGCTGGCAAGGTCCTGCTCCCGGCCCGGGG 159
Cyprinus        GGGCAGAGGAAATATGGTGGTCCTCCTCCGGGCTGGCAAGGTCCTGCTCCCGGCCCGGGC 159
Devario         GGGCAGAGGAAATATGGTGGTCCTCCTCCGGGTTGGCAGGGTCCTGCTCCTGGTCCAGGC 178
Danio           GGGCAGAGGAAATATGGTGGTCCTCCTCCAGGTTGGCAGGGTCCTGCTCCTGGTTCGGGC 240
                ** ***** ** ***** ** ** ***   *  ***   **  *  * ** **  *  *

Oryzias         TGTGAGGTCTTTATCAGCCAGATCCCACGGGATGTCTACGAGGACCTGCTCATCCCCCTC 213
Oncorhynchus    TGTGAGGTGTTCATCAGCCAGATCCCGCGGGATGTCTTTGAGGACCAGCTGATTCCGCTG 216
Carassius       TGTGAGGTTTTCATCAGTCAGATCCCATGTGATGTCTATGAGGACCACCTGATCCCTCTT 219
Cyprinus        TGTGAGGTTTTCATCAGTCAGATCCCATGTGATGTCTATGAGGACTGCCTGATCCCTCTT 219
Devario         TGTGAGGTTTTCATCAGTCAGATCCCACGTGACATCTTCGAAGACCGCCTGATCCCCCTC 238
Danio           TGTGAGGTTTTCATCAGTCAGATCCCGAACGACGTGTACGAGGACCGCCTGATCCCTCTC 300
                ******** ** ***** ********    **  * *  ** ***   ** ** ** **

Oryzias         TTCAGCTCGGTGGGGGCACTTTGGGAATTCCGGCTCATGATGAACTTCAGCGGTCAGAAC 273
Oncorhynchus    TTCCGTGCGGTGGGCCCTCTCTGGGAGTTCCGCCTCATGATGAACTTCAGCGGACAGAAC 276
Carassius       TTCCAGAGCATTGGAACAATTTATGAATTTCGTCTCATGATGAACTTCAGTGGGCAGAAC 279
Cyprinus        TTCCAGAGCATTGGCACTATTTACGAGTTTCGTCTCATGATGAACTTCAGTGGGCAGAAC 279
Devario         TTCCAGAGCGTCGGCACCATTTACGAGTTTCGCCTCATGATGAACTTCAGCGGGCAGAAC 298
Danio           TTCCAGAGCATCGGCACCATTTACGAGTTTCGCCTCATGATGAACTTCAGCGGGCAGACC 360
                ***       * **  *  * *  ** ** ** ***************** ** **** *

Oryzias         CGGGGCTTCGCTTACGCCAAATACGGCACGGCCGCCATCGCCAATGATGCCATCCACCTC 333
Oncorhynchus    CGTGGCTTTGCCTACGCCAAGTACGACAGCCCTGCCTCGGCCGCTGCCGCCATCCGCTCG 336
Carassius       CGTGGCTTCGCCTACGCTAAGTATGGTGACCCGGTCACCGCCTCCGCTGCCGTTATGACC 339
Cyprinus        CGAGGCTTTGCCTATGCAAAGTACGGCGACCCGGTCACCGCCTCCGCTGCCGTCATGACC 339
Devario         CGAGGCTTTGCCTACGCCAAGTATGGTGACCCTCTTACAACCTCGAGAATCTGCCCGCTG 358
Danio           CGGGGCTTCGCCTATGCTAAGTACGGTGACCCTCTTACAGCCTCGGCTGCCGTCACCACG 420
                ** ***** ** ** ** ** ** *      *        **        *

Oryzias         CTTCACGGCTACCCGCTGGGGCCTGGGGCTCGCCTCAGCGTGCGCTGTAGCATCGAGAAG 393
Oncorhynchus    TTGCATGGCCGTGCCCTGGAGTCAGGGGCACGCCTCAGTGTACGGCGCAGCACAGAGAAA 396
Carassius       CTGCATCACTACCGTCTGCCGGAGGGGGGATGCCTGACCGTGCGCAAGAGCACAGAGAAG 399
Cyprinus        CTGCATCACTACCGTCTGCCGGAGGGGGGCTCCCTGACGGTGCGCAAGAGCACAGAGAAG 399
Devario         AAGTTCTCAT-------------------------------------------------- 368
Danio           CTGCATCAGTACCGGCTGCCGGAGGGGGGCTGCCTGACCGTGCGCAGGAGCACCGAGAAG 480
```

Supplementary Figure 5. Alignments of giant danio (*Devario aequipinnatus*) dead-end mRNA partial sequence with other species. The asterisk represents the conserved nucleotides across all species aligned: *Danio rerio*, *Cyprinus carpio*, *Carassius auratus*, *Oryzias latipes* and *Oncorhynchus mykiss*.

Supplementary Note 1

Giant danio *dead-end* gene amplicon sequence (Morpholino target sequence is highlighted with turquoise)

GGAGATGCTCGCAGACATGGAGGCTCAACGGCATTTACAGCAGATTCTGA
ACCCGGTCAAAGTGAAGTCACTGCAGGAATGGATGCAGAAAAACTCCATC
AATTTAACCCAGGTCAACGGGCAGAGGAAATATGGTGGTCCTCCTCCGGG
TTGGCAGGGTCCTGCTCCTGGTCCAGGCTGTGAGGTTTTCATCAGTCAGA
TCCCACGTGACATCTTCGAAGACCGCCTGATCCCCCTCTTCCAGAGCGTC
GGCACCATTTACGAGTTTCGCCTCATGATGAACTTCAGCGGGCAGAACCG
AGGCTTTGCCTACGCCAAGTATGGTGACCCTCTTACAACCTCGAGAATCT
GCCCGCTGAAGTTCTCAT

Sanger's sequencing results for qPCR amplified gene

eukaryotic translation elongation factor 1 alpha 1, like 1 (*eef1a1l1*) 175bp:

TGATCCGCCATTGTTGAGATGGTCCCAGGCAAACCCATGTGTGTGGAGAGCTTCT
CCACCTACCCTCCTCTTGGTCGCTTTGCTGTGCGTGACATGAGGCAGACCGTTGC
CGTTGGTGTCATCAAGAGCGTTGAGAAGAAAGTTGGTGGCAGTGGCAAGGTCACA
AAGTCTGCAA

***Sox9a*_Male (115bp):**

TCAGGCAAAGCGGATCTGAAACGGGAGGCCCGTCCTCTTCAGGAAAACACGGGA
CGTCCGCTCAGCATCAACTTCCAGGACGTGGACATCGGCGAGCTGAGCAGCGAT
GTTATAG

***cyp19a1a*_Female (181bp):**

TTTCATCTGGTCTGGGATCGGGACTGCCAGCAACTACTACAACAACAAATATGGAG

ACATTGTGCGGGTCTGGATCAACGGTGAGGAGACTCTCGTCTTAAGCAGGCCATC

TGCTGTGTATCATGTGTGAGGAAGTCTCTGTACACTTCACGGTTTGGAAGTAAACT

GGGTCTGCAGTGCA

***Vasa* (124bp):**

TGTGCCACCTACCCTGAAGATATTCAAAGAATGGCAGCAGATTTTCTGAAAGTGGA

CTACATTTTCCTTGCTGTTGGTGTGGTGGGTGGAGCATGCAGTGATGTGGAGCAAA

CCGTTGTTCAGA

Supplementary Table 1.

Relative gene expression data ($2^{-\Delta\Delta Cq}$) for MO-treated and control giant danios

	sox9a	*cyp19a1a*	*Vasa (ddx4)*
MO1	0.437291335	0.010796194	0.000201537
MO2	1.461291865	0.029292328	0.000256871
MO3	0.561231024	0.024975661	0.000115753
MO4	1.38943027	0.011651558	0.000124924
MO5	1.009263349	0.019595506	0.000177897
MO6	1.266321368	0.00774063	0.000111037
MO7	1.098341071	0.019325729	0.000281093
MO8	1.004631674	0.032277366	0.000127548
MO9	0.601512518	0.005785537	0.000125792
MO10	1.040596862	0.011023045	0.000147534
GD-F	0.013856115	0.771996743	0.98851402
GD-F	0.012750226	1.501772904	1.605845764
GD-F	0.021443239	0.862542032	1.322560146
GD-M	1.844791174	0.097846677	1.870382496
GD-M	1.63202897	0.038651459	1.819236788
GD-M	1.654811245	0.029701234	0.791868805

MO1- 10 (MO-treated recipients), GD-F (Giant danio control females), and GD-M (Giant danio control males)

Supplementary Table 2.

Fertilization trial with donor-derived sperm.

Group	Total	256-cell stage	25-somite	Day 5
ZFF × GCM1	100	83	78	78
ZFF × GCM2	100	82	80	78
ZFF × GCM3	100	87	87	87
ZFF × GCM4	100	75	67	67
ZFF × GCM5	100	79	73	73
ZFF × ZFM1	100	86	86	86
ZFF × ZFM2	100	79	75	75
ZFF × ZFM3	100	88	72	66
ZFF × ZFM4	100	86	75	75
ZFF × ZFM5	100	85	78	78
ZFF × GDM1	100	12	0	0
ZFF × GDM2	100	3	3	0
ZFF × GDM3	100	6	3	0
ZFF × GDM4	100	2	2	0
ZFF × GDM5	100	5	5	0
GDF × GDM1	100	95	92	92
GDF × GDM2	100	83	83	83
GDF × GDM3	100	86	83	82
GDF × GDM4	100	92	89	89
GDF × GDM5	100	96	91	91
GDF × GCM1	100	10	2	0
GDF × GCM2	100	8	0	0
GDF × GCM3	100	3	3	0
GDF × GCM4	100	11	0	0
GDF × GCM5	100	1	1	0

Supplementary Table 3.

Reproductive performance of germline chimera

	Sperm concentration (×10^4/µl)	Milt volume (in µl)	Total sperm count (×10^4)
GCM-1	25.2	4.2	105.84
GCM-2	11.9	4	47.6
GCM-3	23.8	3.6	85.68
GCM-4	13.2	4	52.8
GCM-5	13.5	3.2	43.2
MEAN± SD	17.52 ± 6.419	3.8 ± 0.4	67.02 ± 27.39
ZF-1	4.5	0.8	3.6
ZF-2	10.2	0.5	5.1
ZF-3	9.2	0.7	6.44
ZF-4	4.7	0.5	2.35
ZF-5	3.9	1	3.9
MEAN± SD	6.5 ± 2.957	0.7 ± 0.21	4.278 ± 1.55
GD-1	30.9	5.4	166.86
GD-2	18.6	4.7	87.42
GD-3	21.3	5.2	110.76
GD-4	16.9	5	84.5
GD-5	19.3	5.6	108.08
MEAN± SD	21.4 ± 5.54	5.18 ± 0.35	111.5 ± 33.11

GCM- Germline chimera male, ZF- Zebrafish, and GD- Giant danio

Supplementary Table 4.

Primers used for the qualitative PCR for MO-treated recipients

Primer name	Sequence (5'-3')
Elf1_F	GATGCCGCCATTGTTGAGAT
Elf1_R	TGCAGACTTTGTGACCTTGC
Sox9a_F	GGGCTGTACTCCACCTTCAG
Sox9a_R	ACTGTGGTTGGATTGAGGGA
cyp19a1a_F	CCTGCAGGAAATGGACAGTG
cyp19a1a_R	TTCACGTTGTAGCCCTCGAT
vasa_F	GTGCCACCTACCCTGAAGAT
vasa_R	CTGAACAACGGTTTGCTCCA
