# Supplementary material for: Enhancement of zebrafish sperm production via a large body-sized surrogate with germ cell transplantation
Source: Commun Biol. 2023 Apr 14;6:412. doi: 10.1038/s42003-023-04800-7 (PMC10104805; doi:10.1038/s42003-023-04800-7)
Supplement: Supplementary file 3 — Reporting Summary [file 42003_2023_4800_MOESM3_ESM.pdf]

nature portfolio

Corresponding author(s): Rigolin Nayak

Last updated by author(s): Mar 22, 2023

Reporting Summary

Nature Portfolio wishes to improve the reproducibility of the work that we publish. This form provides structure for consistency and transparency in reporting. For further information on Nature Portfolio policies, see our Editorial Policies and the Editorial Policy Checklist.

Statistics

For all statistical analyses, confirm that the following items are present in the figure legend, table legend, main text, or Methods section.

n/a	Confirmed	
☐	☒	The exact sample size (*n*) for each experimental group/condition, given as a discrete number and unit of measurement
☐	☒	A statement on whether measurements were taken from distinct samples or whether the same sample was measured repeatedly
☐	☒	The statistical test(s) used AND whether they are one- or two-sided<br>*Only common tests should be described solely by name; describe more complex techniques in the Methods section.*
☐	☒	A description of all covariates tested
☐	☒	A description of any assumptions or corrections, such as tests of normality and adjustment for multiple comparisons
☐	☒	A full description of the statistical parameters including central tendency (e.g. means) or other basic estimates (e.g. regression coefficient) AND variation (e.g. standard deviation) or associated estimates of uncertainty (e.g. confidence intervals)
☐	☒	For null hypothesis testing, the test statistic (e.g. F, t, r) with confidence intervals, effect sizes, degrees of freedom and P value noted<br>*Give P values as exact values whenever suitable.*
☒	☐	For Bayesian analysis, information on the choice of priors and Markov chain Monte Carlo settings
☒	☐	For hierarchical and complex designs, identification of the appropriate level for tests and full reporting of outcomes
☒	☐	Estimates of effect sizes (e.g. Cohen's d, Pearson's r), indicating how they were calculated

Our web collection on statistics for biologists contains articles on many of the points above.

Software and code

Policy information about availability of computer code

Data collection	Data for RT-qPCR was collected from CFX384 Real-Time system (BioRad), and the sperm concentration was assessed using Burker counting chamber (BRAND GMBH + CO.KG, Germany). Microscopic images were taken using Leica Fluorescence Microscope and fluorescence microscope (Olympus BX51)
Data analysis	Data was analyzed using GraphPad prism 9

For manuscripts utilizing custom algorithms or software that are central to the research but not yet described in published literature, software must be made available to editors and reviewers. We strongly encourage code deposition in a community repository (e.g. GitHub). See the Nature Portfolio guidelines for submitting code & software for further information.

Data

Policy information about availability of data

All manuscripts must include a data availability statement. This statement should provide the following information, where applicable:

- Accession codes, unique identifiers, or web links for publicly available datasets
- A description of any restrictions on data availability
- For clinical datasets or third party data, please ensure that the statement adheres to our policy

All supporting data are included in the manuscript and the supplementary files.

Human research participants

Policy information about studies involving human research participants and Sex and Gender in Research.

Reporting on sex and gender	n/a
Population characteristics	n/a
Recruitment	n/a
Ethics oversight	n/a

Note that full information on the approval of the study protocol must also be provided in the manuscript.

Field-specific reporting

Please select the one below that is the best fit for your research. If you are not sure, read the appropriate sections before making your selection.

☒ Life sciences ☐ Behavioural & social sciences ☐ Ecological, evolutionary & environmental sciences

For a reference copy of the document with all sections, see nature.com/documents/nr-reporting-summary-flat.pdf

Life sciences study design

All studies must disclose on these points even when the disclosure is negative.

Sample size	Sample size was determined based on the available literatures for for germ cell isolation and transplantation in other fish.
Data exclusions	No data was excluded from analysis
Replication	Sperm concentration assessment and the fertilization test was replicated according the number of adult germ-line chimera.
Randomization	Donors were randomly chosen for spermatogonia isolation. Giant danio bloodstocks were also randomly selected for recipient production. Gonads from the adult sterile giant danio were randomly selected for histology and gene expression analysis by qPCR.
Blinding	Germline chimera screening and confirmation was performed using all transplant fish. Blinding was used for histology and gene expression analysis for the sterile giant danio.

Reporting for specific materials, systems and methods

We require information from authors about some types of materials, experimental systems and methods used in many studies. Here, indicate whether each material, system or method listed is relevant to your study. If you are not sure if a list item applies to your research, read the appropriate section before selecting a response.

Materials & experimental systems

n/a	Involved in the study	
☐	☒	Antibodies
☒	☐	Eukaryotic cell lines
☒	☐	Palaeontology and archaeology
☐	☒	Animals and other organisms
☒	☐	Clinical data
☒	☐	Dual use research of concern

Methods

n/a	Involved in the study	
☒	☐	ChIP-seq
☒	☐	Flow cytometry
☒	☐	MRI-based neuroimaging

Antibodies

Antibodies used	Anti-DDX4 (ab13840) primary antibody was used for Vasa immunolabeling. Comapny: abcam, host species: Rabit<br>Secondary antibody used: Goat anti-Rabbit IgG (H+L) Cross-Adsorbed Secondary Antibody, Alexa Fluor™ 488 (ThermoFisher Scientific), Catalog # A-11008.
Validation	Anti-DDX4 (ab13840) diluted to 1:300 with 4% goat serum in PBS and secondary antibody (goat anti-rabbit IgG (H+L) Cross-Adsorbed Secondary Antibody, Alexa Fluor™ 488) was diluted to 1:500 with PBS for giant danio gonad

Animals and other research organisms

Policy information about studies involving animals; ARRIVE guidelines recommended for reporting animal research, and Sex and Gender in Research

Laboratory animals	Tg(ddx4:egfp) zebrafish (3 - 4 months), AB zebrafish (age 6 to 7 months) lines and giant danio (age 6 to 7 months) were used in this study.
Wild animals	The study did not involve wild animals.
Reporting on sex	Histological analysis and sex-specific gene expression analysis was performed to identify the sex of sterile giant danio. All the sterile giant danios were found to be phenotypically male and the source data are included in this manuscript.
Field-collected samples	This study did not involve samples collected from the field.
Ethics oversight	The study was conducted at the Faculty of Fisheries and Protection of Waters (FFPW), the University of South Bohemia in Ceske Budejovice, Vodnany, Czech Republic. The facility has the competence to perform experiments on animals (Act no. 246/1992 Coll., ref. number 16OZ19179/2016–17214). The Institutional Animal Care and Use Committee of the FFPW approved the methodological protocol of the current study according to the law on the protection of animals against cruelty (reference number: MSMT-6406/2019–2).

Note that full information on the approval of the study protocol must also be provided in the manuscript.
